# Cell-cell contact-induced gene editing/activation in mammalian cells using a synNotch-CRISPR/Cas9 system

**DOI:** 10.1007/s13238-020-00690-1

**Published:** 2020-01-30

**Authors:** Hongxin Huang, Xin Zhang, Jie Lv, Hongcheng Yang, Xinlong Wang, Shufeng Ma, Ruoyang Shao, Xin Peng, Ying Lin, Zhili Rong

**Affiliations:** 1 School of Basic Medical Sciences, Cancer Research Institute, Southern Medical University, Guangzhou, 510515 China; 2 Dermatology Hospital, Southern Medical University, Guangzhou, 510091 China; 3 Guangzhou Regenerative Medicine and Health Guangdong Laboratory, Guangzhou, 510005 China

**Dear Editor,**

The CRISPR system has been widely used for genome manipulation in various cells, tissues and whole organisms. Although an increasing variety of inducible CRISPR systems have been exploited for a variety of applications, such as chemical switch (Zetsche et al., 2015), photo switch(Shao et al., 2018) and solution ligand switch (Baeumler et al., 2017; Kipniss et al., 2017; Schwarz et al., 2017) systems, a cell-cell interaction inducible system is absent. The synthetic Notch (synNotch) receptor is a recently developed cell-cell contact sensing platform, which contains a customized extracellular sensor module, a transmembrane core domain of native Notch, and a customized intracellular responder module (Morsut et al., 2016). Because of its extraordinary flexibility in terms of customizable sensing/response behaviors, the synNotch receptor serves as a powerful tool for cell engineering (Roybal et al., 2016a; Roybal et al., 2016b; He et al., 2017). In the current study, we combined the synNotch receptor with the CRISPR/Cas9 system to develop a cell-cell interaction inducible gene regulated tool.

Since the previously reported synNotch receptor was based on mouse Notch1 (M1) (Morsut et al., 2016), we tried to develop other synNotch receptors using different Notch family members from several species, including human (H), mouse (M), drosophila (Fly) and zebrafish (Z), with anti-CD19-ScFv/mCherry as a sensor/responder module (Fig. 1A). We found that the new synNotch receptors demonstrated better activation than the M1 synNotch (especially for Z3, 78.0% ± 9.8% of cells were activated with a 158.4 ± 19.7-fold change), while showing a background ranging from 0.5% to 45% (Figs. 1B, 1C and S1A). M4 system was not activated when treated with CD19+ Cells (Figs. 1B and S1A), which was consistent with the previous report that Notch4 does not signal in response to ligand but inhibits signaling from the Notch1 receptor (James et al., 2014). To decrease the background noise, P2A-Gal4KRAB or P2A-Gal4 was added downstream of Gal4-VP64 (Fig. S1B). Gal4KRAB completely blocked activation (Fig. S1C), and Gal4 dramatically decreased the background noise (M2, ~10%; all the others, <5%) but simultaneously attenuated activation significantly (all, <15%) (Fig. S1D). It has been reported that EGF (epidermal growth factor) repeats can prevent the constitutive activation of Notch (Sakamoto et al., 2005). Therefore, we included an extra EGF repeat on the extracellular domain between the anti-CD19 ScFv and the Notch core domain (Fig. S2A). By including an extra EGF, the background in the Z1, Z2, and Z3 systems was decreased, and nearly eliminated in the Fly system (Fig. S2B). However, it also affected activation, as represented by the Z3 system with an efficiency of decreasing to approximately 41.2% when stimulated (Fig. S2B and S2C). To balance background and efficiency, we shortened the EGF repeat by half (eZ3), which remarkably increased the stimulation efficiency to 62.8% while maintaining a tolerable background (8.5%) (Fig. S2B and S2C).

**Figure 1:**
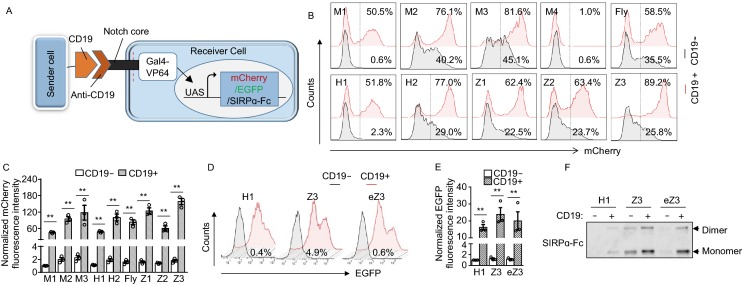
**Various activities of novel synNotch receptors based on Notch family members in different species**. (A) Diagram of the synNotch system. (B) Flow cytometry analyses showed the activation levels of mCherry in the new synNotch systems. The receiver cells were U2OS cells with a mCherry reporter. The sender cells were K562 cells with/without CD19. Red: CD19+, black: CD19−. M: mouse Notch, H: human, Fly: drosophila, Z: zebrafish. (C) Normalized fluorescence intensity of mCherry in flow cytometry (*n* = 3, Student’s *t* test, **P < 0.01, error bars, SEM). (D) Activation of EGFP in H1, Z3 and eZ3 systems. (E) Normalized fluorescence intensity of EGFP in flow cytometry (*n* = 3, Student’s *t* test, **P < 0.01, error bars, SEM). For (D) and (E), the receiver cells were MCF7 cells with an EGFP reporter. (F) Western blot showed the secretion of the SIRPɑ-Fc protein in the H1, Z3 and eZ3 systems. The receiver cells were MCF7 cells with a downstream SIRPɑ-Fc

Next, we tested the ability of the synNotch receptors to respond to different sender cells and the response flexibility of the receiver cells. In addition to K562-CD19 cells, CD19-transduced B16 melanoma cells (B16-CD19) with either high (B16-10) or low (B16-3) CD19 expression could activate mCherry expression in the M1 and H1 systems (Fig. S3A and S3B). However, mouse splenocytes expressing mouse CD19 failed to activate the M1 and H1 receptors, demonstrating the specificity of the synNotch systems (Fig. S3C and S3D). To test the flexibility of the response modules in the synNotch systems, we replaced the mCherry reporter with EGFP and SIRPɑ-Fc in the H1, Z3 and eZ3 systems (Fig. 1A). FACS and fluorescence microscopy analyses showed that EGFP was potently activated by the CD19+ cells (Figs. 1D, 1E and S4). High-affinity SIRPɑ-Fc (CV1-hIgG4) holds great therapeutic potential and has entered a phase I clinical trial for solid tumor treatment (Weiskopf et al., 2013). SIRPɑ-Fc also could be secreted by the receiver cells triggered by CD19+ sender cells (Fig. 1F).

To develop a cell-cell contact-induced gene editing system, we swapped the Gal4-VP64 domain into Cas9 (SpCas9). In HEK293T-dEGFP cells that stably express a fast-degradable EGFP variant, transfected synNotch-Cas9 and EGFP-targeting sgRNA could down-regulate EGFP even without stimulation by CD19+ cells, suggesting a severe signal leakage (Fig. S5A). Split Cas9 with C-terminus and N-terminus fusing to FKBP and FRB, respectively, induces gene editing upon rapamycin treatment (Zetsche et al., 2015). We then swapped the Cas9 into split N-Cas9 and found the leakage issue remained (Fig. S5B). Next, the linkers between Notch core and Cas9/N-Cas9 were optimized by replacing with Linker 7/8 (L7/L8) or adding Linker 7/8 (Q + L7/Q + L8). FACS results showed that the regulation by CD19+ cells was improved, but the leakage issue still existed (Fig. S5C). Together, the strategy of direct fusing split Cas9/Cas9 to synNotch receptors required further optimization.

Since the direct fusion approach did not work well, we tried another strategy by substituting the responder gene with a Cas9:p300 fusion protein to establish a synNotch-Cas9:p300 system (Fig. 2A). The T2A-puro-UAS-Cas9:p300 fragment was knocked-in at the *AAVS1* locus in Jurkat cells through homology-independent targeted integration (HITI)(Suzuki et al., 2016) (Fig. S6A). The validated knocked-in clone A28 (Fig. S6B) was transduced with the synNotch (H1) receptor (Fig. S6C). Western blot analysis showed that Cas9:p300 could be up-regulated by CD19+ cells (Fig. S6D). When transfected with 14-bp sgRNAs, the expression level of *MYOD* was up-regulated significantly with CD19+ cell stimulation (Fig. S6E). To test the potential of the system for application in immunotherapy, several immune genes were chosen for further investigation. PD-1 and CTLA4 are two checkpoint molecules that play pivotal roles in suppressing the activity of T cells and two major targets for cancer immunotherapy. IL-2 supports the survival and proliferation of T cells and CCL19 recruits T cells, and both molecules have been reported to increase the efficacy of T cell-based cancer immunotherapy (Rosenberg, 2014; Adachi et al., 2018). In the H1 synNotch-Gal4UAS-Cas9 system, *PD-1* and *CTLA4* were edited, and *CCL19* and *IL2* were up-regulated only when exposed to the CD19+ cells and in the presence of 20-bp and 14–15-bp sgRNAs, respectively (Figs. 2B, 2C and S7A). Since the Z3 system demonstrated the highest activation in all the tested systems (Fig. 1), we replaced H1 with Z3 in the synNotch-Cas9:p300 system. As predicted, the activation of *CCL19* and *IL2* and the editing of *PD-1* and *CTLA4* were elevated; however, the background noise was also increased (Fig. S8A and S8B). Thus, the synNotch-Gal4UAS-Cas9:p300 systems could induce gene editing and activation via cell-cell interaction, a local microenvironment-controlled manner.

**Figure 2:**
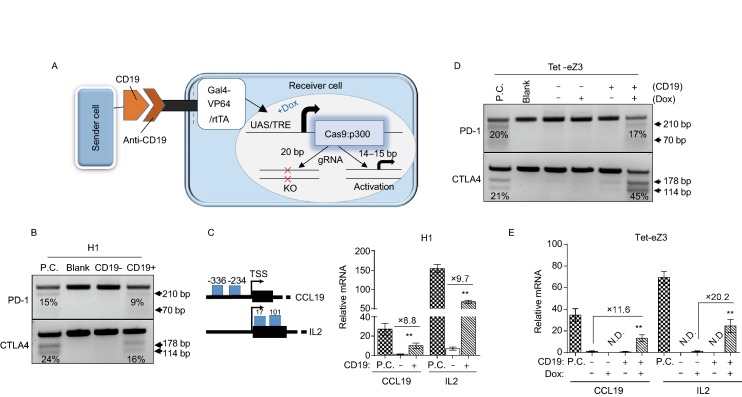
**SynNotch-Cas9:p300 systems edit and activate immune genes with 20 bp- and 14–15 bp-length sgRNAs, respectively**. (A) Diagram of the synNotch-Cas9:p300 system. (B and C) Editing of *PD-1* and *CTLA4* (B) and activation of *CCL19* and *IL2* (C) with the synNotch-Cas9:p300 system (H1) was detected through T7E1 and q-PCR assays, respectively. The numbered blue boxes indicate the binding regions of the 14**–**15 bp sgRNAs. (D and E) Editing of *PD-1* and *CTLA4* (D) and activation of *CCL19* and *IL2* (E) with the synNotch-Tet-Cas9:p300 system (eZ3) was examined. For the q-PCR: the results of one representative experiment out of three repeats are shown. Student’s *t* test, **P < 0.01, error bars, SEM. N.D., not detected. P.C., Jurkat cells transfected with plasmids encoding a constitutively expressed Cas9:p300 and the corresponding gRNAs. Blank, Jurkat cells

To develop a more precisely controlled device, we introduced the Tet-on system to generate a synNotch-Tet-Cas9:p300 system which was controlled by both CD19+ cells and Dox (Doxycycline) treatment (Fig. 2A). The T2A-puro-TRE-Cas9:p300 cassette was knocked-in at the *AAVS1* locus through HITI (Fig. S9A) and the validated knocked-in clone C2 was transduced with the eZ3 system to establish stable cell lines (Fig. S9B and S9C). The expression of the Cas9:p300 fusion protein and *MYOD* was up-regulated only when both CD19+ cells and Dox were applied (Fig. S9D and S9E). *PD-1* and *CTLA4* were edited and *CCL19* and *IL2* were significantly up-regulated only in the presence of both CD19+ cells and Dox (Figs. 2D, 2E and S7B). Therefore, the alternative system synNotch-Tet-Cas9:p300 could control gene regulation via a spatiotemporal manner, giving that cell-cell contact provided a spatial control and that the time of adding Dox provided a temporal control.

In this work, we have developed new synNotch receptors with distinct activation and background features and combine two of them with CRISPR/Cas9 to generate a synNotch-Cas9 system which can edit or activate endogenous genes in a cell-cell-contact-controlled manner. Compared with previously reported systems, such as CRISPR ChaCha (Kipniss et al., 2017), dCas9-synR (Baeumler et al., 2017) and MESA(Schwarz et al., 2017), our synNotch-Cas9 system performs with distinguished properties. First, the triggers of the synNotch-Cas9 system are the surface-bonded ligands (particularly, cell surface ligands) rather than the solution ligands. Cell-cell contact systems provide more precise location control, enabling site-specific activation, while soluble ligand systems enable long-range communication between cell populations via diffusion. Second, the synNotch-Cas9 system is based on the synNotch receptor, which is extraordinarily programmable in engineering cells with customized sensing/response behavior. The reported MESA, dCas9-synR, and CRISPR ChaCha systems can currently only detect natural ligands, and thus lead to the problem of orthogonality, the engineered pathways interfering with endogenous pathways. Third, the synNotch-Cas9 system can both edit and activate endogenous genes simultaneously. Extracellular signals induce the expression of Cas9:p300, which edits and activates genes when bonded with 20-bp and 14–15-bp sgRNAs, respectively. Thus, the synNotch-Cas9 system provides an alternative, complementary strategy for cell engineering, which might be a powerful tool to investigate physiological and pathological processes, including mapping cell interactions during development and engineering therapeutic cells for disease treatments.

## Electronic supplementary material

Below is the link to the electronic supplementary material.

Supplementary material 1 (PDF 1227 kb)

Supplementary material 2 (XLSX 10 kb)

Supplementary material 3 (XLSX 10 kb)

Supplementary material 4 (XLSX 10 kb)
