# Supplementary material for: Cell-cell contact-induced gene editing/activation in mammalian cells using a synNotch-CRISPR/Cas9 system
Source: Protein Cell. 2020 Jan 30;11(4):299–303. doi: 10.1007/s13238-020-00690-1 (PMC7093359; doi:10.1007/s13238-020-00690-1)
Supplement: Supplementary file 1 — Supplementary material 1 (PDF 1227 kb) [file 13238_2020_690_MOESM1_ESM.pdf]

Methods

Plasmid construction

The synNotch system plasmids pHR-PGK-antiCD19-synNotch-Gal4VP64 (#79125), pHR-Gal4UAS-IRES-mCherry-pGK-BFP (#79123) and the Split-Cas9 plasmids pLSC-5 (#62889) were purchased from Addgene. To construct the new synNotch receptors, notch core sequences from different species, which were cloned from tissues or cells from mouse, human, fly and zebrafish, were inserted into a common vector, pHR-PGK-antiCD19-BamHI/XhoI-Gal4VP64, which was derived from the original synNotch plasmid (#79125). The PA2-Gal4-KRAB/Gal4 sequences were added behind Gal4-VP64 via Gibson Assembly. The downstream response plasmids lenti-Gal4UAS-EGFP and lenti-Gal4UAS- SIRPα-Fc were constructed via Gibson Assembly using a lenti-backbone vector (Addgene, #52962), Gal4UAS, EGFP and SIRPα-Fc fragments. To generate the synNotch-Cas9 system, the antiCD19- Notch(M1)-QLCIQKL linker was amplified from the synNotch plasmid (#79125) and fused to Cas9 expression vector by Gibson Assembly. For the synNotch-spilt-Cas9 system, the NES-N Cas9-FRB from pLSC-5 (#62889) was cloned to the synNotch-Cas9 plasmid to replace Cas9, and the NLS-FKBP-C Cas9 was ligated to a pBlueScript backbone with a CAG promoter and a SV40 polyA signal sequence. The linker 7 and linker 8 were synthesized and sub-cloned into the synNotch-Cas9 system to replace or extend the original linker. The donor plasmid

pBlu-AAVS1-TRE-Cas9:p300-AAVS1 was derived from a pBlu-AAVS1-TRE-Cas9:p300-M2rtTA-AAVS1 plasmid(Ma et al., 2018) by deleting the M2rtTA fragment. The donor plasmid pBlu-AAVS1-UAS-Cas9:p300-AAVS1 was constructed by replacing the TRE (tetracycline responsive element) element with the Gal4UAS fragment (PCR amplified from the Addgene plasmid, #79123) in pBlu-AAVS1-TRE-Cas9:p300-AAVS1. Long (20 bp) and short (14-15 bp) sgRNAs targeting the genes of interest were synthesized, annealed and ligated to the plasmid expressed U6+sgRNA$^{(F+E)}$ (Chen et al., 2013). All primers are listed in Table S1. All sgRNA and the Linker 7 and 8 sequences are listed in Table S2. All constructs were verified through Sanger sequencing.

Cell culture

The K562 and B16 sender cells were transduced to stably express human CD19 and a BFP reporter. The U2OS receiver cells were first transduced with the Gal4UAS-IRES-mCherry-pGK-BFP response element, then a single clone was infected with lentiviruses encoding synNotch receptors. For EGFP or SIRPα-Fc protein activation, the MCF7 receiver cells were transduced with both the Gal4UAS-EGFP or Gal4UAS-SIRPα response element and the synNotch receptor antiCD19-Notch-Gal4-VP64 lentivirus in a pool mix. In the synNotch-Cas9:p300 system, the Jurkat receiver cells underwent knock-in with the Gal4UAS/TRE-Cas9:p300 response element via HITI (Suzuki et al., 2016; Ma et al., 2018) and were then transduced with H1/Z3/Tet-eZ3 synNotch

receptors to establish stable cell lines. The genotyping primers to identify the correct knock-in clones are listed in Table S3.

K562, B16, and U2OS cells were maintained in Dulbecco's Modified Eagle's Medium (DMEM, Life Technologies) at 37°C in a 5% CO2 humidified incubator. MCF7 and Jurkat cells were cultured in RPMI 1640 medium (Life Technologies) at 37°C in a 5% CO2 humidified incubator. All growth media were supplemented with 2 mM L-Glutamine (Life Technologies), 100 U/mL penicillin, 100 μg/mL streptomycin (Life Technologies), and 10% FBS.

Lentivirus production

All lentiviruses were produced by co-transfecting the transfer plasmids, and package and envelope plasmids (pMD2.G and psPAX2) using the PEI reagent (Sigma, USA) in HEK293-FT cells plated in 6-well plates at approximately 75% confluency. Viral supernatants were collected 3 days after transfection and then filtered using a 0.45 μm filter (Millipore, USA). The supernatant was used for transduction immediately or kept at -80°C for long-term storage.

Activation of synNotch and synNotch-Cas9:p300 systems

For synNotch system activation, 3×10^5 sender cells were co-cultured with receiver cells which were plated in 24-well plates a day before at a 1:1 ratio. After approximately 48 h, the receiver cells were analyzed using flow cytometry (BD, USA). Fluorescence images were obtained using a confocal microscope (Nikon, Japan). For the synNotch-Cas9:p300 system, 3×10^5 B16 sender cells were plated in 24-well plates, and the next day, after

electroporation with the targeting sgRNAs using the Amaxa Cell Line Nucleofector Kit V (VCA-1003, LONZA, Switzerland) in accordance with the manufacturer's instructions (2D, Program X-001), the Jurkat receiver cells were added to the B16 or B16-CD19 for approximately 2 d. Finally, aliquots of suspension of Jurkat cells were used for western blotting, T7E1, and q-PCR analyses.

T7E1 and TIDE analyses

For T7E1 analysis, the amplicons were purified, denatured at 95°C for 5 min and annealed in NEB Buffer 2 with a slow ramp down (approximately −2°C/min) to 4°C, then subjected to T7 endonuclease I (NEB, UK) digestion for 3 h at 37°C before loading on a 2% agarose gel. The primers for T7E1 to amplify the amplicons were listed in Table S3.

The Tracking of Indels by Decomposition (TIDE) method (Brinkman et al., 2014) was applied for analyzing the editing indels and determining their frequencies in a cell population using Sanger sequences of the PCR amplicons from control samples and testing samples. All the parameters were set to the default and the TIDE analysis tool is available online (http://tide.nki.nl). The primers for TIDE were as the same as that used for T7E1.

Quantitative real-time PCR

Total RNA from the receiver cells was isolated using Trizol Reagent (Thermo Fisher, USA) in accordance with the manufacturer's instructions. Total RNA (1

μg) was reverse transcribed into cDNA and then quantitative real-time PCR (SYBR Premix Ex Taq II, TAKARA, China) was performed using a LightCycler 96 System (Roche, Switzerland). Relative gene expression was calculated using the $2^{-\Delta\Delta Ct}$ method after normalizing to GAPDH expression. All the qPCR primers are listed in Table S3.

Western blotting

To detect the SIRPα-Fc secretory protein, sender cells and receiver cells were co-cultured in DMEM without FBS for approximately 2 d. Then the supernatant media was subjected to 5× SDS loading buffer without reducing agents and boiled for 5 minutes. The lysates were resolved through SDS/PAGE and transferred onto a nitrocellulose membrane which was blocked using 5% non-fat milk, and sequentially incubated with HRP-conjugated goat anti-human IgG secondary antibody (Bioss, China). The probed proteins were finally detected through chemiluminescence in accordance with the manufacturer's instructions (Pierce, USA).

To detect the Cas9:p300 protein, the co-cultured receiver cells were lysed in 2× SDS loading buffer and boiled for 10 min. The lysates were resolved through SDS/PAGE and transferred onto a nitrocellulose membrane which was blocked using 5% non-fat milk and sequentially incubated with primary antibodies

(anti-flag, sigma, USA; anti-b-tubulin, Proteintech, China) and a HRP-conjugated horse anti-mouse IgG secondary antibody (CST, USA). All

the probed proteins were finally detected through chemiluminescence in accordance with the manufacturer’s instructions (Pierce, USA).

FACS analyses

All flow cytometry analyses were performed using FlowJo software (TreeStar, USA). To compare the activation level of the synNotch systems, sender cells were gated out using BFP, and the receiver cells were gated using mCherry or GFP. The activation efficiency was determined as the proportion of mCherry or GFP positive cells within the total receiver cells, and the activation fold change was the ratio of the mean fluorescence intensity between positive and negative background cells.

References

Brinkman, E.K., Chen, T., Amendola, M., and van Steensel, B. (2014). Easy quantitative assessment of genome editing by sequence trace decomposition. Nucleic Acids Res 42, e168.

Chen, B., Gilbert, L.A., Cimini, B.A., Schnitzbauer, J., Zhang, W., Li, G.W., Park, J., Blackburn, E.H., Weissman, J.S., Qi, L.S., *et al.* (2013). Dynamic imaging of genomic loci in living human cells by an optimized CRISPR/Cas system. Cell 155, 1479-1491.

Ma, S., Lv, J., Sun, J., Tang, P., Li, H., Zhou, H., Zhang, Z., Lin, Y., and Rong, Z. (2018). iKA-CRISPR hESCs for inducible and multiplex orthogonal gene knockout and activation. 592, 2238-2247.

Suzuki, K., Tsunekawa, Y., Hernandez-Benitez, R., Wu, J., Zhu, J., Kim, E.J., Hatanaka, F., Yamamoto, M., Araoka, T., Li, Z., *et al.* (2016). In vivo genome editing via CRISPR/Cas9 mediated homology-independent targeted integration. Nature 540, 144-149.

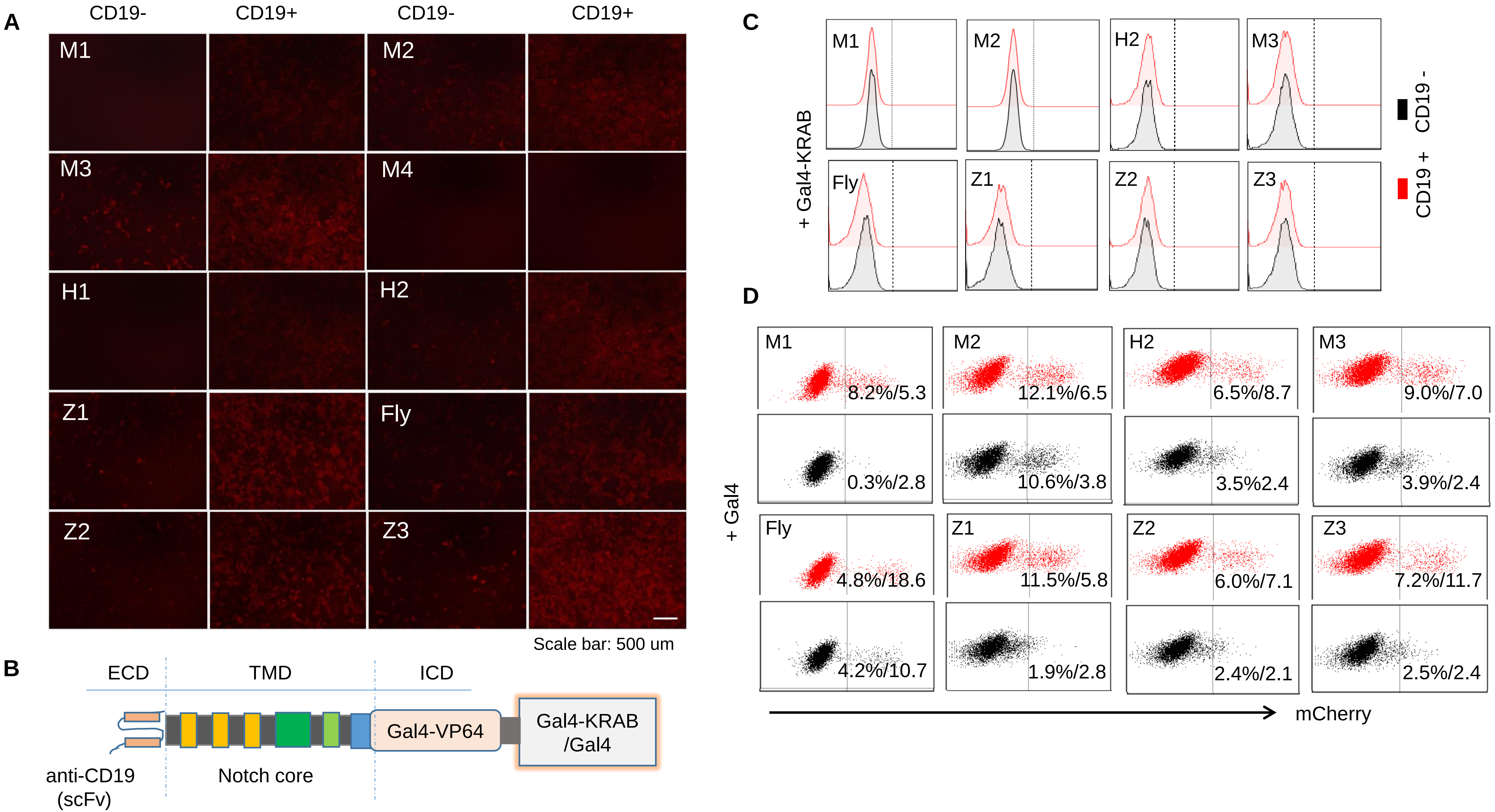

Figure S1. Decreasing the basal activation of synNotch systems by adding Gal4KRAB or Gal4.

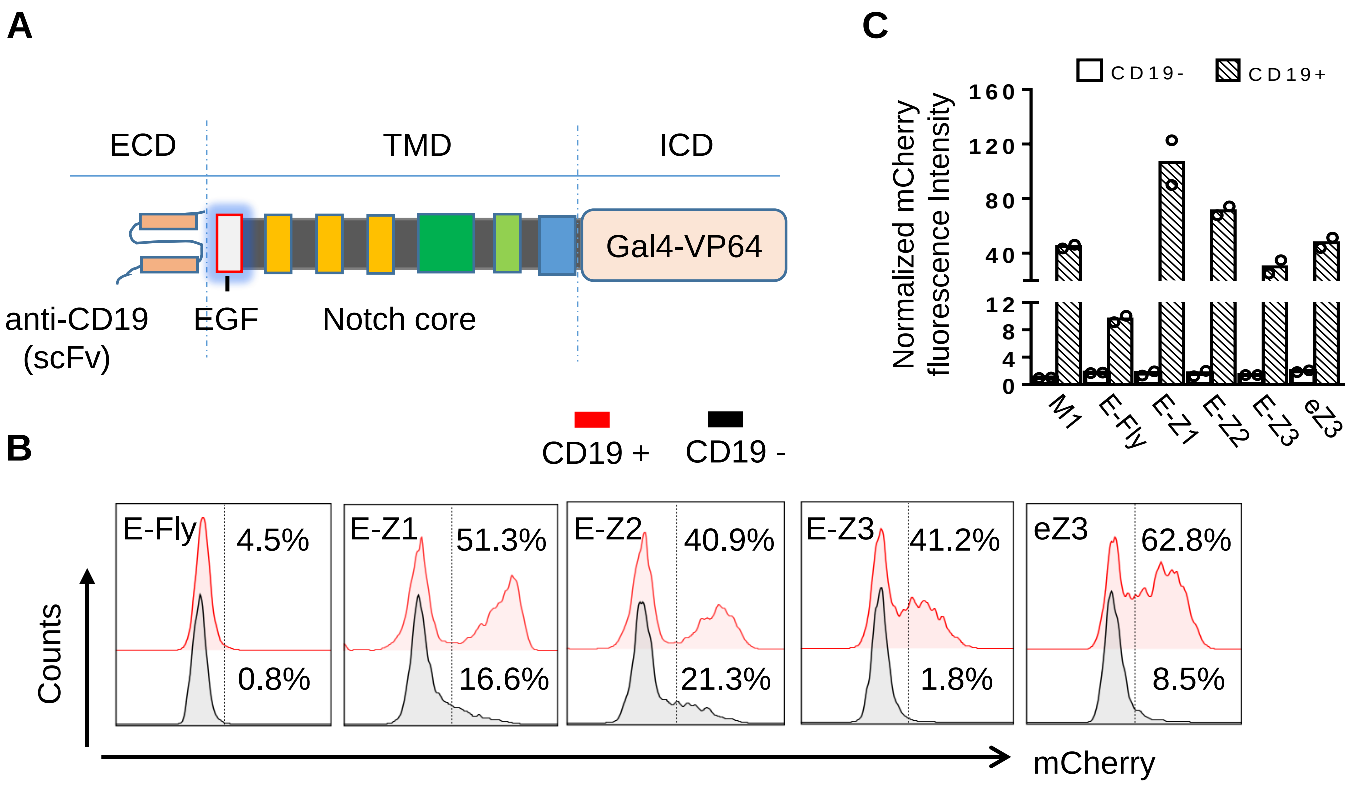

Figure S2. Decreasing the basal activation of synNotch systems by extending EGF domains.

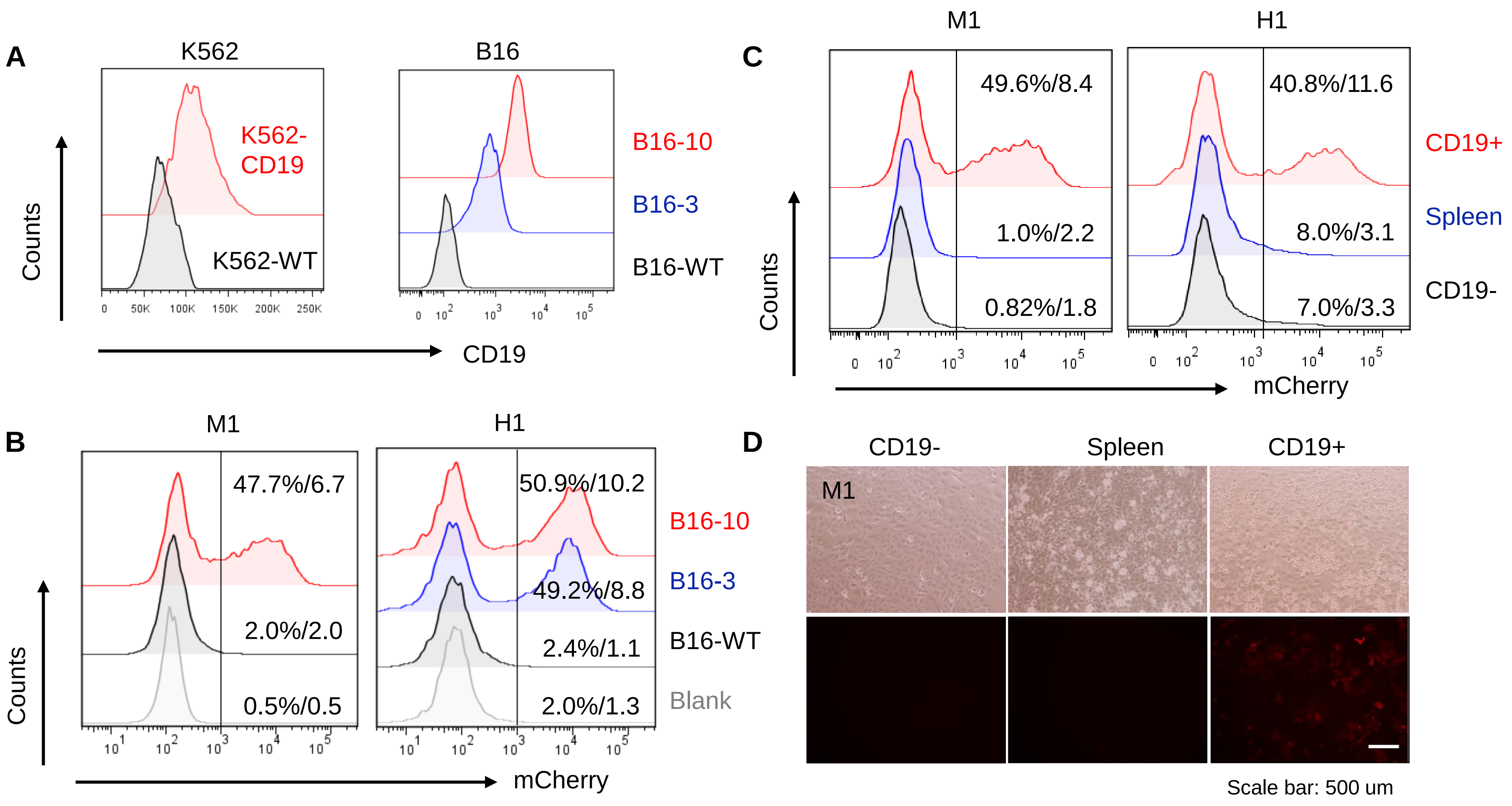

Figure S3. Activation of the synNotch systems with different sender cells.

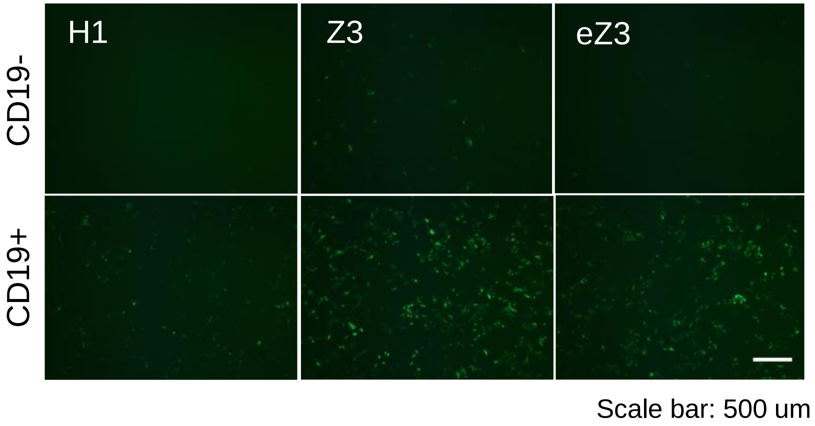

Figure S4. Activation of EGFP with H1, Z3 and eZ3 systems.

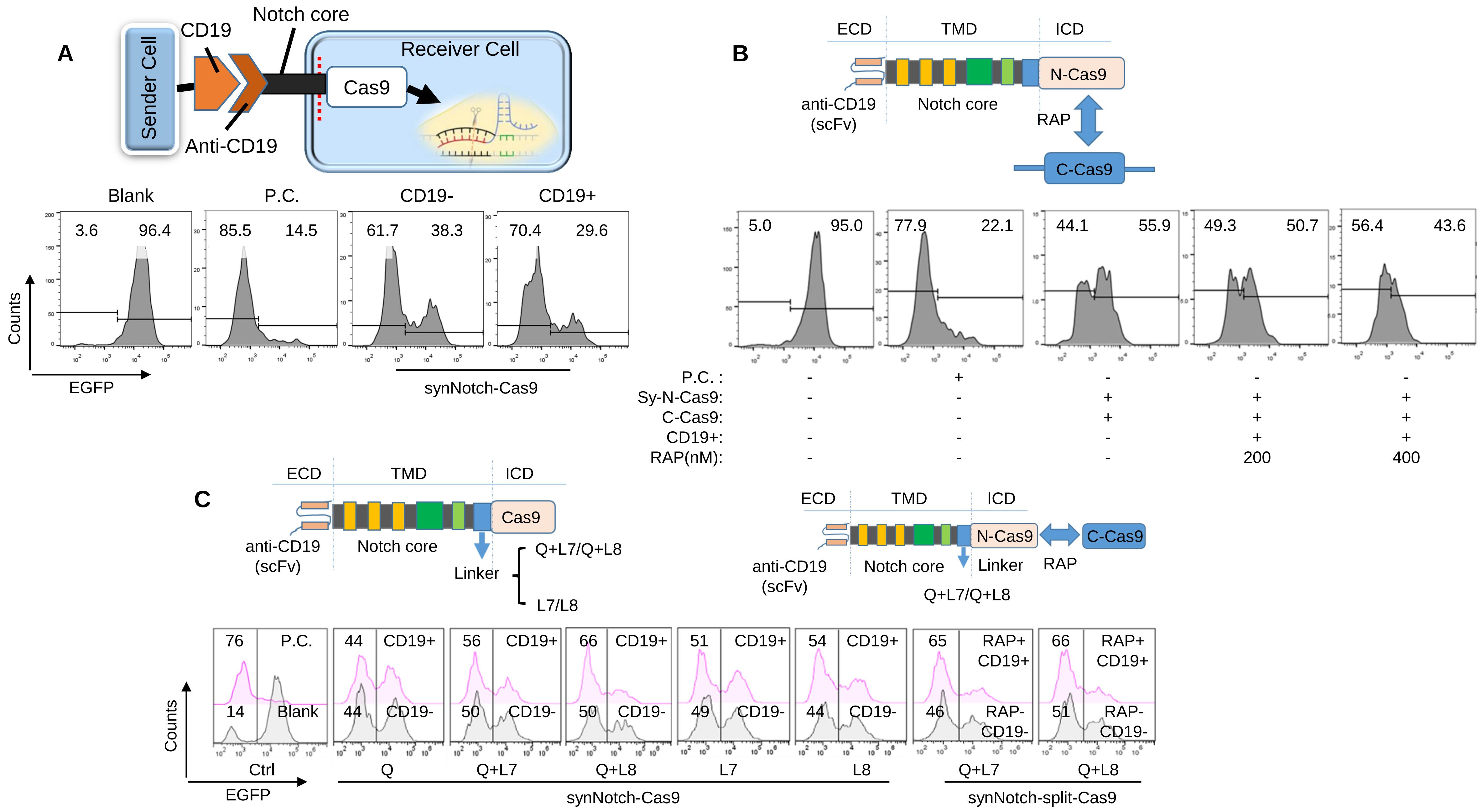

Figure S5. The direct fusion strategy to develop the synNotch-Cas9 system.

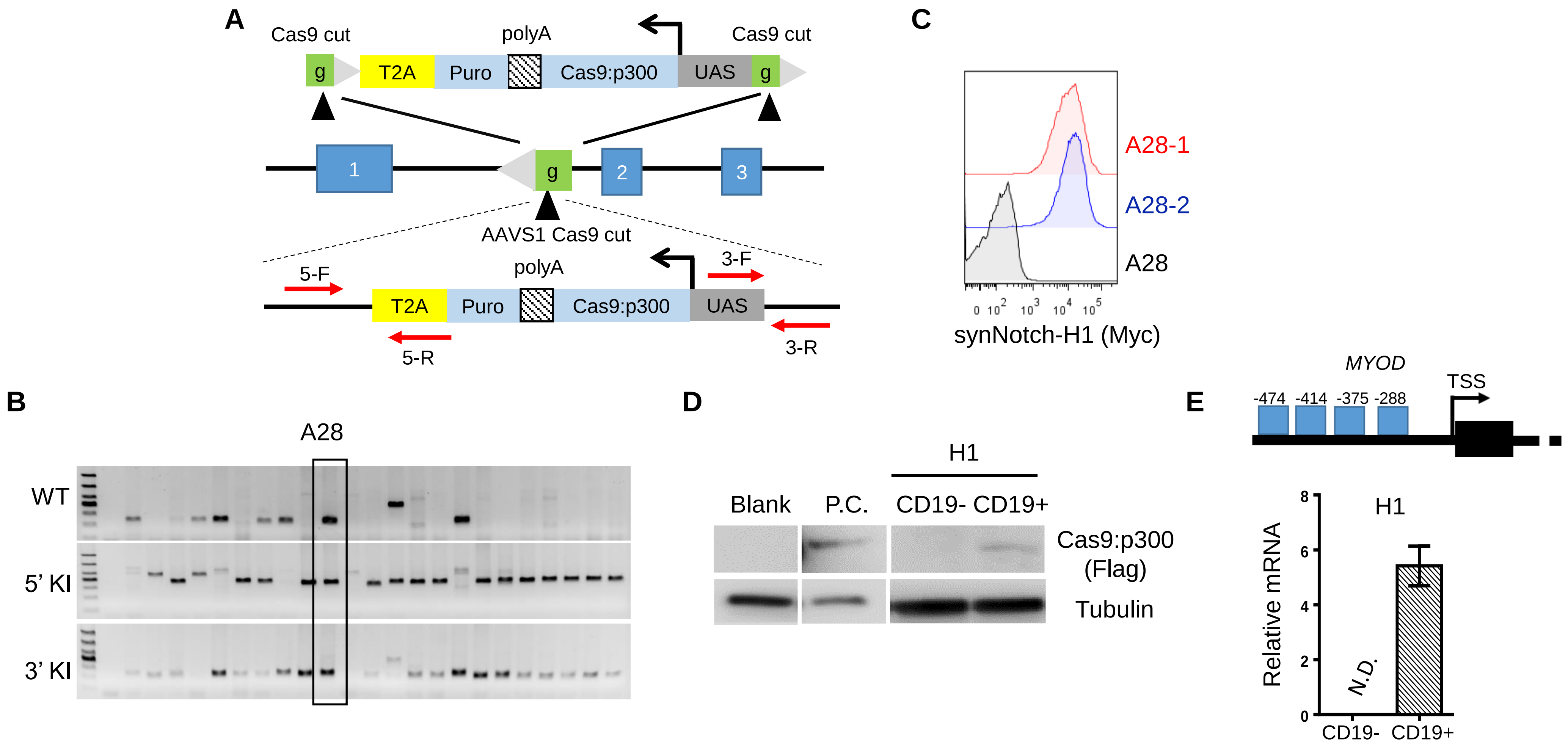

Figure S6. Construction of synNotch-Gal4/UAS-Cas9:p300 Jurkat cell lines

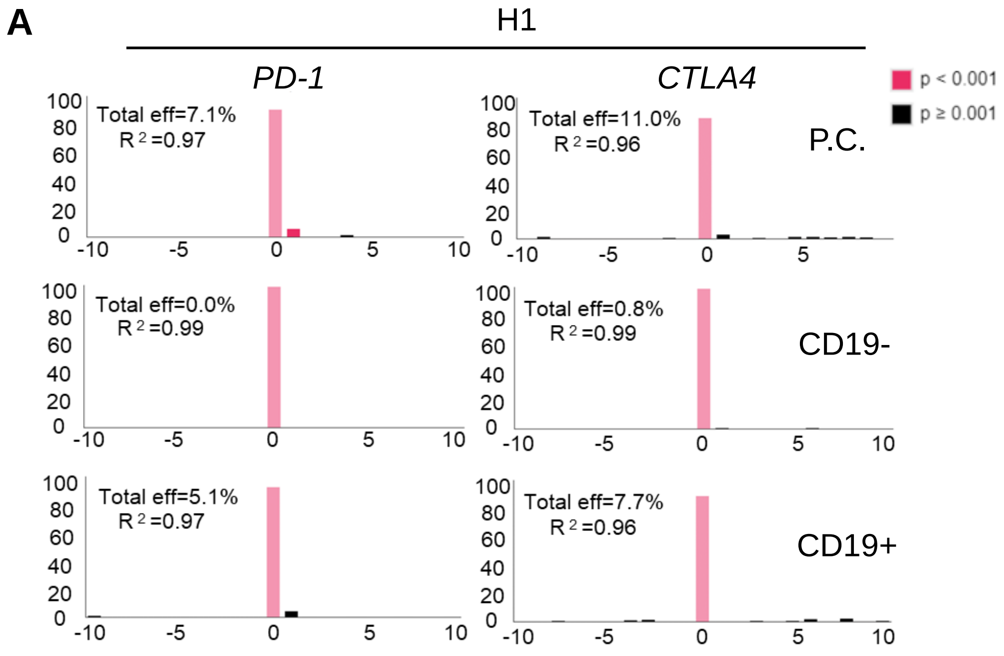

TIDE analyses (H1)

	PD-1	*CTLA4*
P.C.:	7.1%	11.0%
CD19-:	0.0%	0.8%
CD19+:	5.1%	7.7%

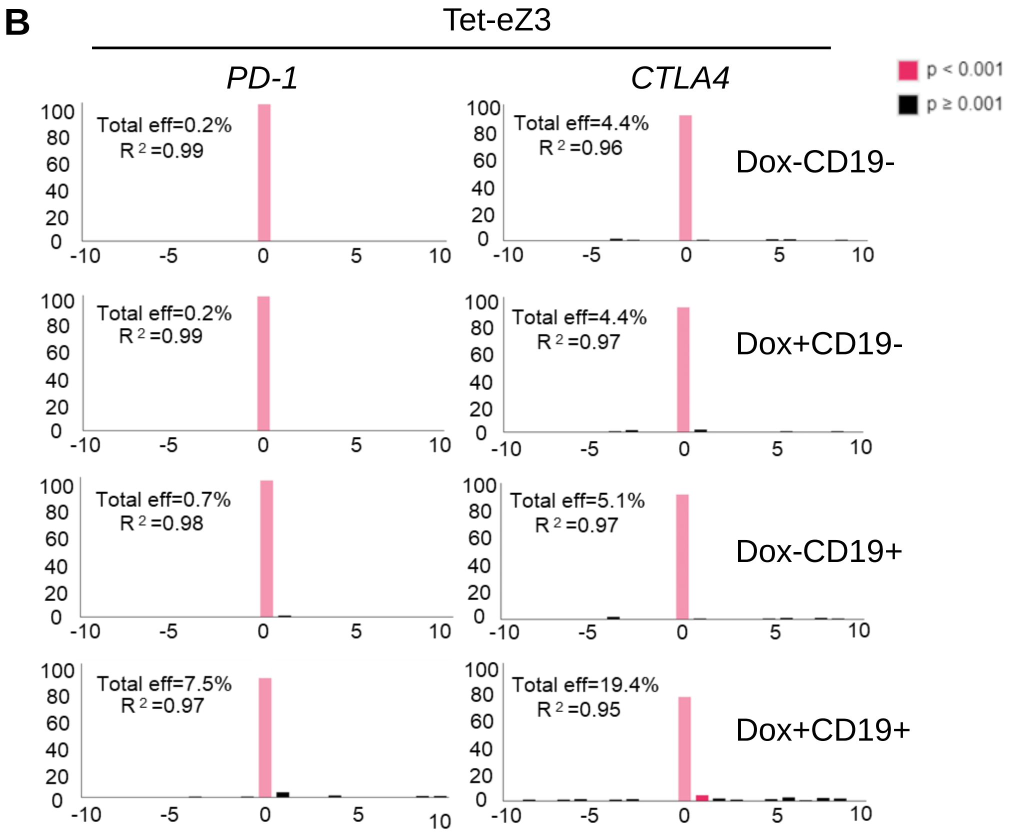

TIDE analyses (Tet-eZ3)

	PD-1	*CTLA4*
Dox-CD19-:	0.2%	4.4%
Dox+CD19-:	0.2%	4.4%
Dox-CD19+:	0.7%	5.1%
Dox+CD19+:	7.5%	19.4%

Figure S7. The editing efficiencies of *PD-1* and *CTLA4* in the synNotch-Cas9 systems using TIDE assay.

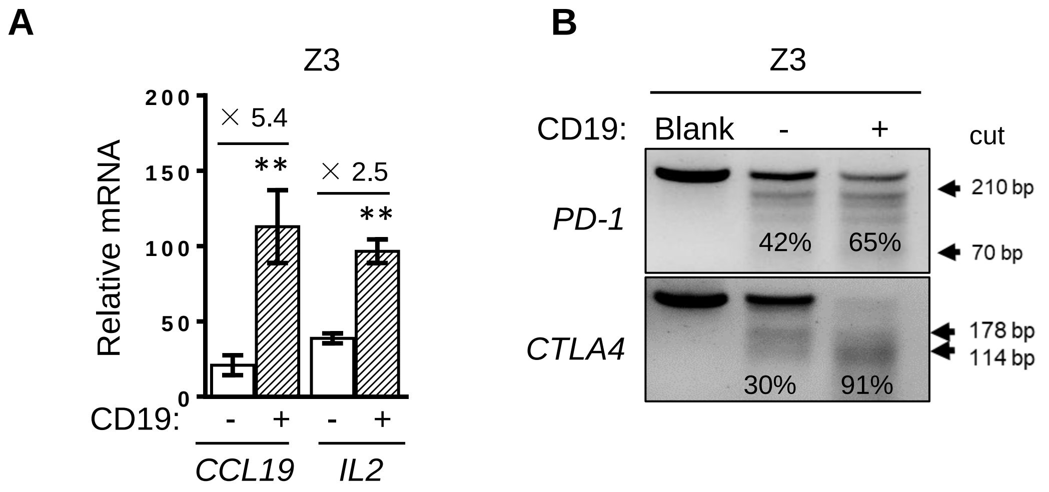

Figure S8. SynNotch-Cas9:p300 systems can activate and edit the endogenous genes by sgRNAs with different length.

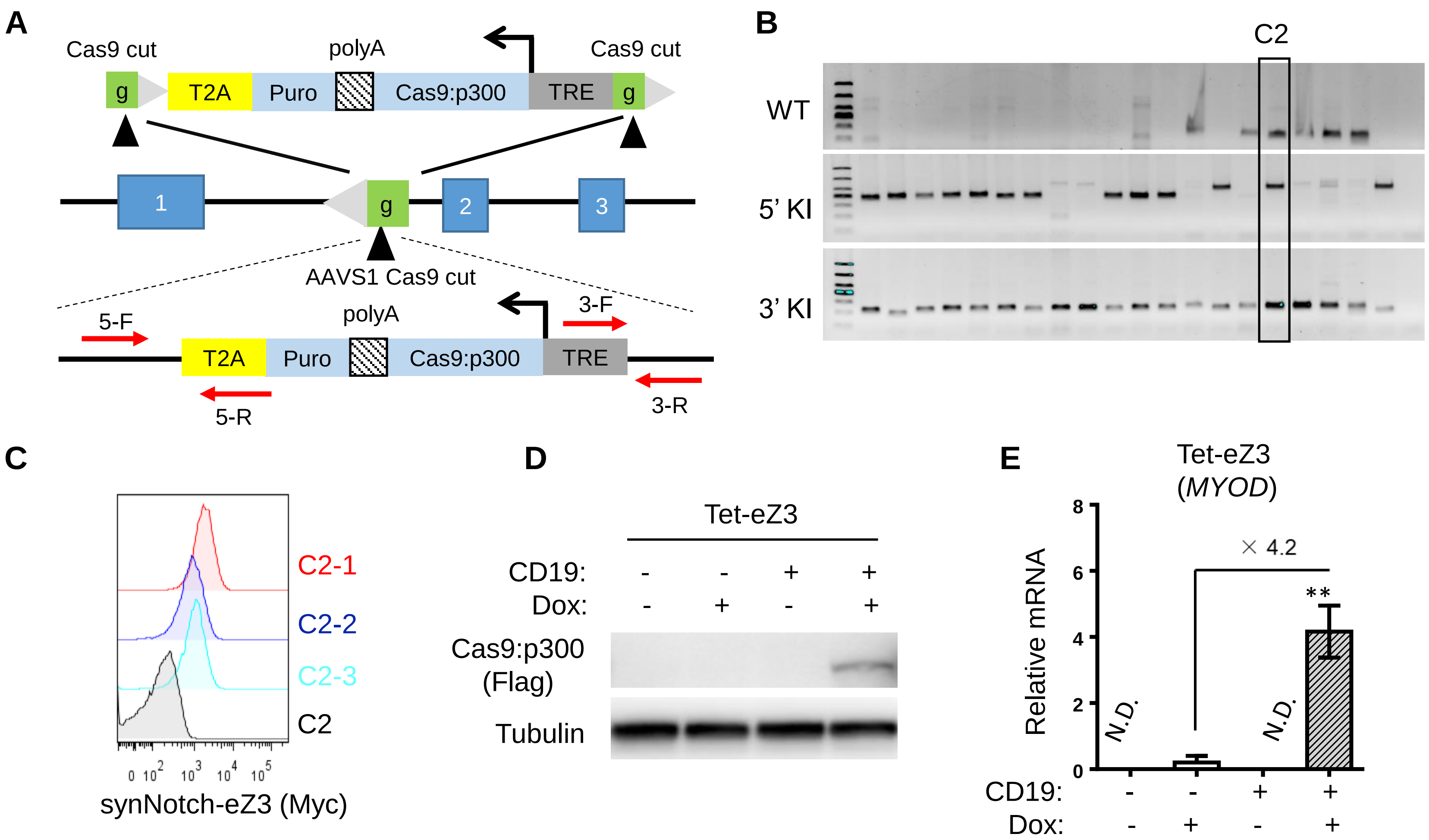

Figure S9. Construction of synNotch-Tet-Cas9:p300 Jurkat cell lines.

Figure S1. Decreasing the basal activation of synNotch systems by adding Gal4-KRAB or Gal4. (A) Microscopy analyses of mCherry expression in different versions of the synNotch system. Scale bar: 500 μm. (B) Diagram of the synNotch receptor with Gal4-KRAB or Gal4. P2A-Gal4-KRAB/Gal4 was added downstream of Gal4-VP64 to compete with the UAS to reduce basal activation. ECD, extracellular domain. TMD, transmembrane domain. ICD, intracellular domain. (C) Gal4-KRAB completely blocked synNotch receptor activation. U2OS receptor cells engineered with synNotch-Gal4-KRAB failed to activate the mCherry reporter upon stimulation with CD19+ K562 sender cells. Red: CD19+, black: CD19-. (D) Gal4 decreased basal activation. U2OS receptor cells expressing synNotch-Gal4 receptors responded with decreased background and reduced activation (compared with that shown in Fig.1B). Percent/value, activation efficiency/mean fluorescence intensity (×1000, a.u.).

Figure S2. Decreasing the basal activation of synNotch systems by extending EGF domains. (A) Diagram of the synNotch receptor with extended EGF repeats. (B) An extra EGF repeat decreased basal activation. eZ3, the Z3 receptor with an extra half EGF repeat. (C) Normalized fluorescence intensity of mCherry in the EGF-synNotch systems.

Figure S3. Activation of the synNotch systems with different sender cells. (A, B) B16 cells with different CD19 expression levels could activate the synNotch systems. Clone B16-10 expressed CD19 at a high level compared with clone B16-3. Percent/value, activation efficiency/mean fluorescence intensity (×1000,

a.u.). (C, D) FACS (C) and Microscopy analyses (D) showed mouse spleen cells failed to activate the human CD19-scFv synNotch system. Scale bar: 500 μm.

Figure S4. Activation of EGFP with H1, Z3 and eZ3 systems. Microscopy analyses of EGFP expression in the H1, Z3 and eZ3 synNotch systems. Scale bar: 500 μm.

Figure S5. The direct fusion strategy to develop the synNotch-Cas9 system. (A) The CD19-scFv:Notch core:Cas9 synNotch receptor (synNotch-Cas9) displayed high background leakage. (B) The synNotch-split-Cas9 system maintained high background. (C) Linker optimization of the synNotch-Cas9 and synNotch-split-Cas9 systems. For (A-C), HEK293T-dEGFP reporter cells were transfected with the synNotch-Cas9, synNotch-NCas9/pCAG-C-Cas9, linker optimized plasmids, and an EGFP-targeting sgRNA plasmid, and stimulated with CD19- or CD19+ cells. P.C., cells transfected with a plasmid encoding a constitutively expressed Cas9. RAP: rapamycin. Q: the original linker QLCIQKL amplified from the synNotch plasmid (#79125), L7/L8: two linkers for optimization with sequences listed in Table S2, Q+L7/Q+L8: linkers with both Q and L7/L8.

Figure S6. Construction of synNotch-Gal4/UAS-Cas9:p300 Jurkat cell lines. (A) The T2A-puro-UAS-Cas9:p300 fragment knock-in strategy. The T2A-puro element driven by the endogenous promoter of *PPP1R12C* upstream of the

AAVS1 site was used for single clone selection. A sgRNA recognition site in the reverse direction was ligated at each end of the targeting vector. Primers for genotyping are indicated as red arrows (5-F, 5-R, 3-F, and 3-R). (B) Identification of the correct knock-in single Jurkat cell clones through PCR. A28 was one of the correct clones. (C) Cell surface expression of the synNotch receptor (H1) in synNotch-Gal4/UAS-Cas9:p300 stable cell lines as assessed using FACS. A28 clone cells were transduced with the H1 receptor (fused with a Myc tag at the N-terminus) to construct synNotch-Gal4/UAS-Cas9:p300 (H1) cells. (D) Western blotting assay showed the inducible expression of the Cas9:p300 fusion protein (3× Flag tag) in the responder cells by CD19+ cell stimulation. P.C., a positive cell lysate transfected with constitutively expressed Cas9:p300. (E) q-PCR assays showed that the *MYOD* gene was activated with 14-bp sgRNAs in the synNotch-UAS/Cas9:p300 system (H1). The numbered blue boxes indicate the binding sites of the 14-bp sgRNAs. TSS, transcription start site. N.D., not detected.

Figure S7. The editing efficiencies of *PD-1* and *CTLA4* in the synNotch-Cas9 systems using TIDE assay. (A) *PD-1* and *CTLA4* were edited upon stimulation with the CD19+ cells in the synNotch-Cas9:p300 systems (H1). (B) *PD-1* and *CTLA4* were edited in the presence of both CD19+ cells and Dox in the synNotch-Tet-Cas9:p300 system (eZ3). P.C., Jurkat cells transfected with plasmids encoding a constitutively expressed Cas9:p300 and the corresponding sgRNAs.

Figure S8. SynNotch-Cas9:p300 systems can activate and edit endogenous genes by sgRNAs with different length. (A, B) q-PCR and T7E1 assays showed that *CCL19* and *IL2* were activated with 15-bp or 14-bp sgRNAs and *PD-1* and *CTLA4* were edited with 20-bp sgRNAs in synNotch-UAS/Cas9:p300 (Z3) responder cells with high background noise. For q-PCR assays: Representative results of one from three repeated experiments are shown. Student's t test, $^{**}p<0.01$, error bars, SEM.

Figure S9. Construction of synNotch-Tet-Cas9:p300 Jurkat cell lines. (A) The T2A-puro-Tet-Cas9:p300 fragment knock-in strategy, which is similar to the strategy shown in Fig. S6A. (B) Identification of the correct knock-in single Jurkat cell clones using PCR. C2 was one of the correct clones. (C) Cell surface expression of the synNotch receptor (eZ3) in synNotch-Tet-Cas9:p300 stable cell lines, as assessed using FACS. (D) Western blotting assay showed the inducible expression of the Cas9:p300 fusion protein (3× Flag tag) by CD19+ cell stimulation in the presence of Dox (2 μg/mL). (E) q-PCR assay showed that the *MYOD* gene was activated with the synNotch-Tet-Cas9:p300 system (eZ3). One representative from three repeated experiments are shown. Student's t test, $^{**}p<0.01$, error bars, SEM.
